# Grass and tree cover responses to intra-seasonal rainfall variability vary along a rainfall gradient in African tropical grassy biomes

**DOI:** 10.1038/s41598-019-38933-9

**Published:** 2019-02-20

**Authors:** Donatella D’Onofrio, Luke Sweeney, Jost von Hardenberg, Mara Baudena

**Affiliations:** 1 Institute of Atmospheric Sciences and Climate, National Research Council (ISAC-CNR), Corso Fiume 4, 10133 Torino, Italy; 2 Copernicus Institute of Sustainable Development, Environmental Science Group, Utrecht University, Princetonlaan 8a, 3584 CB Utrecht, The Netherlands

## Abstract

Although it is well known that mean annual rainfall (MAR) and rainfall seasonality have a key role in influencing the distribution of tree and grass cover in African tropical grassy biomes (TGBs), the impact of intra-seasonal rainfall variability on these distributions is less agreed upon. Since the prevalent mechanisms determining biome occurrence and distribution change with MAR, this research investigates the role of intra-seasonal rainfall variability for three different MAR ranges, assessing satellite data on grass and tree cover, rainfall and fire intervals at a sub-continental scale in sub-Saharan Africa. For MAR below 630 mm y^−1^, rainfall frequency had a positive relationship with grass cover; this relationship however became mostly negative at intermediate MAR (630–1200 mm y^−1^), where tree cover correspondingly mostly increased with rainfall frequency. In humid TGBs, tree cover decreased with rainfall intensity. Overall, intra-seasonal rainfall variability plays a role in determining vegetation cover, especially in mesic TGBs, where the relative dominance of trees and grasses has previously been largely unexplained. Importantly, the direction of the effect of intra-seasonal variability changes with MAR. Given the predicted increases in rainfall intensity in Africa as a consequence of climate change, the effects on TGBs are thus likely to vary depending on the MAR levels.

## Introduction

Grasslands and savannas, known together as tropical grassy biomes (TGBs), are estimated to cover ca. a third of Africa and a fifth of the world’s land area^1,2^. Characterised by continuous C_4_ grassland, and possible coexistence with trees^2^, they support a growing proportion of the world’s population, are home to the majority of the world’s remaining megafauna^2^ and are a critical store of biodiversity^3^. Despite their importance, TGBs have garnered little public attention or conservation effort in comparison to tropical forests^1,4^, and the role of grass in characterizing these biomes has only recently been studied at sub-continental level^5^.

The relative dominance of trees and grasses in TGBs and the extent of those systems are governed by numerous feedbacks between bottom-up factors (*sensu* ref.^4^), such as water availability and soil type, and by top-down factors, such as fire and herbivory (ref.^6^ and references therein). Mean annual rainfall (MAR) has been identified as the most important factor, both impacting the availability of water and seemingly influencing the relative importance of other factors^5,7–9^. Whereas tree and grass cover is limited primarily by water availability in dry TGBs, in mesic areas the high fire frequency, fostered by high grass cover, helps to limit tree cover^5,7^. At these higher MAR levels, both tropical forests and savannas exist^10–12^, with savannas mostly observed in areas with highly seasonal rainfall regimes, but partly also under overlapping climatic conditions with forests^5,11,13^. In these areas TGBs are probably maintained by a grass-fire feedback, whereby grasses provide fuel for fire, which in turn restricts the recruitment and growth of shade-tolerant and fire-intolerant forest trees, thus supporting the growth of grasses and fire-resistant savanna trees^14^. Generally, for mesic savannas, the ability to predict the relative dominance of tree and grass cover remains limited^5^.

In TGBs, competition for water by trees and grasses leads to water resource partitioning by root depth^15^, with root-depth separation between trees and grasses observed in dry areas^16,17^. Variations in rooting depth by vegetation type have been demonstrated in TGBs across differing rainfall gradients, with tree roots becoming shallower with MAR and overlapping with grass roots at higher rainfall levels^18^.

Soil water content depends not only on yearly rainfall levels or on rainfall seasonality, but also on the temporal distribution of rainfall within a season, described here as rainfall event frequency and intensity^19^. The intra-seasonal temporal distribution of rainfall can influence vegetation productivity dramatically^20,21^, especially in dry areas^22–24^, and it can affect the outcome of competition between plants with different water strategies^25–27^. Mirroring changes observed in global precipitation variability^28^, changes in rainfall patterns have been observed across Africa over several decades in the last century, with, for example, increases in rainfall intensity in Southern and Western Africa^29,30^, reductions in rainfall frequency specifically in Botswana^31^, decreases in intensity in Central Africa^32^, and increases in drought length across Eastern Africa^33^. Changes in rainfall variability have been closely associated with anthropogenic climate change^34^, and, with rainfall intensity in Africa expected to increase in the future^35^, changes in vegetation patterns may result.

Although a substantial body of research now exists in relation to TGBs and the role of various factors in explaining their occurrence and extent, the relative importance of intra-seasonal rainfall variability is less clearly understood. Recent research on the impact of intra-annual variability of rainfall on tree cover for different MAR ranges in Africa^9^ and across tropical areas^36^ has shown that intra-annual variability has an important role in determining tree cover variation, particularly within the intermediate (500–1500 mm y^−1^) MAR levels^36^. However, the influence of intra-seasonal variability on tree cover depends upon MAR throughout tropical Africa^36,37^, specifically for TGBs^9^. In drier TGBs, tree cover can increase with rainfall intensity^9^, in line with experimental work^38^ showing that increased precipitation intensity increases deep soil water, enhancing woody plant growth; this is supported by the deeper roots observed for trees than for grasses in drier savannas^17,18,39^. These findings are corroborated by modelling work^25^ that includes competition of grasses with tree seedlings, suggesting that more rare and intense rainfall events support the growth of woody plants in arid savannas, due to the out-competition of grasses on tree seedling, an effect which is especially strong at low moisture availability^40,41^. In contrast, areas with lower tree cover in African TGBs have been associated with greater rainfall intensity (although the precise impact depends also on soil type)^9^ which is analogous to that reported by ref.^42^ with data across all African biomes. This finding has been modelled and explained by the different relative-growth rates of grasses and trees^43^ (*sensu* ref.^44^), with grasses having the ability to grow faster than trees in the presence of high soil moisture, but being more negatively impacted under high water stress.

Within this context, the main research question addressed in this paper is whether changes in intra-seasonal rainfall variability affect the propensity of grasses, in addition to trees, to grow in TGBs at different MAR ranges. Given the importance of grasses in determining TGB extent and existence^1^ and their important role in the competition for water with trees^2^, we explicitly included grass cover as a dependent variable, unlike previous, similar studies that analysed large scale satellite data and considered only the tree cover variable^9,36,42^. In so doing we consider the interaction between vegetation types in TGBs at a sub-continental scale. Following a methodological approach closely aligned to that of ref.^5^, which analysed grass cover for the first time at sub-continental scale, we analysed the impact of intra-seasonal temporal variability of rainfall on grass and tree cover at sub-continental scale throughout TGBs in sub-Saharan Africa, in comparison to the impact of fire, MAR and rainfall seasonality (analysed in ref.^5^). As in that earlier study, we assessed separately three different MAR ranges, since different explanatory variables are relevant for low (less than 630 mm y^−1^), intermediate (between 630 mm y^−1^ and 1200 mm y^−1^), and high MAR (above 1200 mm y^−1^). The analysis by MAR range enhances the ability to identify the factors, in addition to rainfall totals, that can explain differences in tree and grass cover, potentially identifying a role for intra-seasonal variability that may have otherwise been overlooked.

## Material and Methods

### Satellite data

We analysed gridded data at 0.5° resolution of observed percentages of tree (T) and grass (G) cover in areas of tropical grassy biomes in sub-Saharan Africa (between 35°S and 15°N) as a function of four different rainfall variables and average fire interval data (AFI, [y]). The rainfall variables were mean annual rainfall (MAR, [mm y^−1^]), average rainfall seasonality index (SI) and two variables representing intra-seasonal rainfall variability: average wet-season daily rainfall frequency (λ_w_, [d^−1^]) and intensity (α_w_, [mm d^−1^]). All variables were averaged over the period 2000–2010. Vegetation cover, fire intervals, mean annual rainfall and rainfall seasonality were calculated following ref.^5^, and as detailed below.

We used the daily rainfall measurements of the tropical rainfall measuring mission (TRMM 3B42), with 0.25° original resolution, to derive MAR, SI^45^, α_w_ and λ_w_ ^24^. As in ref.^5^ we considered grid cells with MAR up to 2500 mm y^−1^. SI is defined by Walsh & Lawler as a ratio with the absolute value of the sum of the differences between monthly rainfall and average monthly rainfall at the numerator and annual rainfall at the denominator. It can range from 0 (if the annual rainfall is equally distributed over the year) to 1.83 (if all the annual rainfall falls in one month). In seasonal environments, such as those associated with savannas, precipitation affects plants mainly during the growing period^46,47^. For this reason, the estimation of daily rainfall intensity and frequency over the wet seasons is more appropriate than over the year. We thus assumed that the wet seasons correspond to the growing seasons of both plant types (although this may not be strictly true for all savannas, see refs^2,48^ and references therein). For each grid cell, α_w_ was computed as the mean, over the years, of the daily rainfall in the wet days (assumed as days with precipitation greater than 0.1 mm d^−1^) within the wet periods (identified as explained in the following paragraph). The small threshold was introduced to take into account observation measurement errors. For each grid cell, λ_w_ was calculated as the ratio of the average number of wet days to the average length of the wet periods (L_w_ [d], calculated as explained in the following paragraph). α_w_ and λ_w_ were defined such that the following relation was verified: MAR_w_ = α_w_·λ_w_·L_w_ + c, where MAR_w_ is the wet-season mean annual rainfall and c is the sum of the rainfall occurring in the wet-season days in which rainfall is smaller than 0.1 mm d^−1^; across the data analysed, c was 0.30 mm y^−1^ on average. Consequently, MAR_w_, L_w_, α_w_ and λ_w_ are not independent of each other.

To calculate the wet-season length L_w_, we identified the wet periods as the months in which mean precipitation was greater than a percent threshold p_thr_ of the climatological annual average monthly rainfall. Then, by summing up the lengths in days of these wet months and dividing by the number of years, we obtained the average wet season length L_*w*_. To decide upon the threshold p_thr_, we maximised the correlation of L_w_ with the length of the wet season calculated with an alternative method, which was related to the opposite of SI (L_w,SI_), similarly to that used by ref.^42^, i.e.: L_w,SI_ = 365/12 [11·(1 − SI/1.83) + 1], so that L_w,SI_ is equal to ca. 30 d for SI_max_ = 1.83 and to 365 d for SI_min_ = 0 ^45^. We chose p_th*r*_ = 50%, as for this value the two measures of wet season length were highly correlated (R^2^ = 0.96).

We obtained the annual average fire interval (AFI) from the monthly MCD45A1 burnt area satellite product (Collection 5.1), with original 500 m resolution, available from April 2000^49–51^. AFI is the expected return time of fire at any point in the 0.5° grid cell^52^, calculated as the inverse of the average annual burnt area fraction (BA, [y^−1^]) in each 0.5° grid cell (AFI = 1/BA). BA was obtained following the method used by ref.^53^. In the analysis we used log_10_(AFI) (equal to −log_10_(BA)), because AFI covered several orders of magnitude. In order to avoid infinity values of AFI (when BA = 0), we set the maximum of AFI to 10,000 years adding to BA a small constant (0.0001 y^−1^) (see also ref.^5^).

Percentages of tree cover (T) and grass cover (G) were obtained from the annual tree and non-tree vegetation cover products, respectively, of the Terra MODIS Vegetation Continuous Fields product (MOD44B, V051), with original 250 m resolution^54^. Since MODIS tends to underestimate tree cover in the presence of shrub^55^, we used the 300-m ESA global land cover map (ESA CCI-LC, v 1.6.1; 5-year-averaged dataset centred in 2010) to remove grid cells with more than 50% of the area covered by shrublands (ESA CCI-LC codes 120,122). We used the same land cover map to mask out grid cells with more than 33% of the area influenced by humans, such as cropland/urban areas, and/or covered by water (codes ≤40, 190, 210) and/or covered by permanent snow or ice (code 220). This conservative choice, motivated by the inclusion of areas with only minimal human impact, selected a representative range of areas in sub-Saharan Africa. See also ref.^5^ for more details.

In order to study the effect of the abiotic variables specifically on tropical grassy biome vegetation, we identified grid cells of TGBs as those with more than 50% of the area flagged on the ESA CCI-LC map as deciduous trees and grassland (codes 60–62,130), following ref.^5^. This choice was based on the fact that African savannas trees are mostly deciduous (refs^56,57^, and see literature review in ref.^5^). The use of this method for distinguishing TGBs avoided problems in identifying biomes based on tree cover discontinuities in MODIS data^58^. The final datasets consisted of 1692 grid cells.

### Statistical modelling

**Figure 1:**
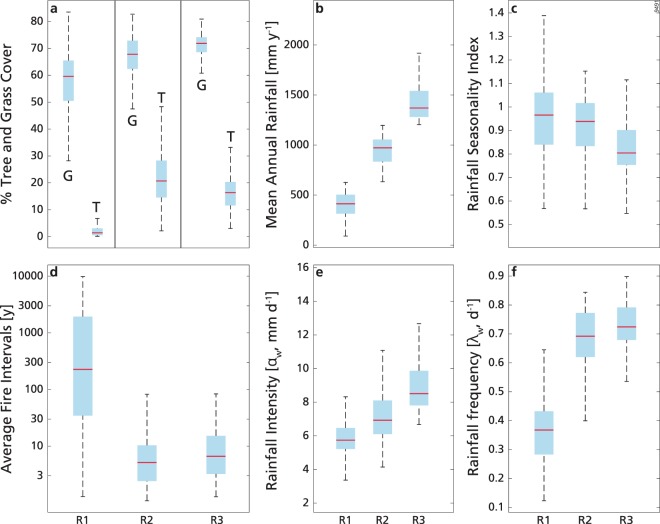
Box plots of percentage cover of tree (T) and grass (G) (**a**), mean annual rainfall (**b**), rainfall seasonality index (**c**), average fire intervals (**d**), wet-season daily rainfall intensity (**e**) and wet-season daily rainfall frequency (**f**), in the low (R1; MAR ≤ 630 mm y^−1^), intermediate (R2; 630 mm y^−1^ < MAR < 1200 mm y^−1^) and high (R3; MAR ≥ 1200 mm y^−1^) MAR ranges. Outliers are not shown. All the distribution medians differed significantly.

We analysed the relationships between vegetation cover variables (T and G) and five abiotic variables (MAR, SI, λ_w_, α_w_ and log_10_(AFI)) using generalized linear models (GLM)^59^, in three different MAR ranges: 0–630 mm y^−1^ (R1), 630–1200 mm y^−1^ (R2) and 1200–2500 mm y^−1^ (R3). These have been determined in the study of ref.^5^ and highlight how MAR influences the role of the prevalent mechanisms related to water availability and fire in determining biome occurrence and tree to grass dominance in sub-Saharan Africa. See a map of their spatial distribution in Supplementary Fig. S1. Figure 1 shows the variable distributions in these three ranges. For completeness, we also repeated the GLM analysis for all the TGB data, without filtering by MAR ranges. Notice that we did not include MAR_w_ and L_w_ in the statistical analysis because MAR_w_ was highly correlated with MAR (R^2^ > 0.98 for each of the three ranges), and L_w_ was anti-correlated with SI (R^2^ > 0.94 for each of the three ranges). In R1, R2 and R3 there were 694, 783 and 215 grid cells, respectively. We tested that the medians of the variable distributions were statistically different at the 5% significance level using the two-sided Wilcoxon rank sum test.

Tree and grass cover fractions were fitted assuming a binomial error distribution with a logit link function, because they are bounded between 0 and 1^60,61^. We investigated the relationships considering models with different terms of a single predictor up to the third order, and models with a combination of the predictors but including only linear terms. Predictor variables were standardized subtracting their mean and dividing their standard deviation, such that their coefficient magnitude was a measure of their importance in the model. Since the rainfall variables used were not independent of each other (see previous section), we decided to exclude models containing both α_w_ and λ_w_. We included also the intercept-only model (i.e., a constant model), obtaining a set of 34 possible models. Models were selected based on the Akaike’s information criterion (AIC)^62^, such that the best models were those with the lowest AIC value, and we reported the ΔAIC (i.e. the difference between the AIC value of each model with the best model). We analysed all models with AIC smaller than the model including the intercept-only. The goodness-of-fit was evaluated as the fraction of deviance explained (pseudo-R^2^, R^2^ in the following for brevity), equivalent to the explained variance in a linear least-squares regression model^63^. It was computed as R^2^ = 1 − D_m_/D_0_, where D_m_ is the residual deviance, i.e. the deviance that remains unexplained by the fit, and D_0_ is the deviance of the intercept-only model^61,63^.

To take into account collinearity between explanatory variables (see Supplementary Table S1 for the Pearson’s r value of the variables in each of the three ranges), we performed a residual analysis for the cases where the best model included α_w_ or λ_w_. This analysis was aimed at understanding whether the influence of these predictors on the vegetation cover was independent of the other explanatory variables. To this end, we computed the GLM of each intra-seasonal rainfall variable (α_w_ or λ_w_) as a function of MAR, SI and log_10_(AFI) (see also Supplementary Note S1 for details), and computed the deviance residuals. We calculated also the deviance residuals of the vegetation cover GLM computed with respect to the same three explanatory variables (as obtained from the above-described analyses). We then calculated the R^2^ of the linear fit of these two residual sets, as a high R^2^ implied that the dependence of the vegetation cover variable on α_w_ or λ_w_ was direct and not only mediated by the other variables.

Data analysis was performed using MATLAB R2015b. In particular, we used the Matlab function ‘fitglm’, for the GLM analysis, and ‘ranksum’ for the Wilcoxon rank sum test.

## Results

In each MAR range, one of the intra-season rainfall variables (α_w_ or λ_w_) always appeared in the best model for either T or G (or both). Yet, when performing the analysis without distinguishing between MAR ranges (i.e. analysing all the dataset at once), the intra-seasonal rainfall variables did not appear in any of the best models: according to these models T depended on MAR and G on both SI and log_10_(AFI) (see Supplementary Tables S7 and S8). In the following, we report the results of the statistical analysis computed in each MAR range separately.

### MAR ≤630 mm y^−1^

For low precipitation areas, T was mainly determined by MAR, while λ_*w*_ and SI were both included in the best model for G (Table 1).

**Table 1:** Best GLMs for tree cover (T) and grass cover (G) in the three mean annual rainfall (MAR) ranges: low MAR (R1, MAR ≤ 630 mm y^−1^), intermediate MAR (R2, 630 mm y^−1^ < MAR < 1200 mm y^−1^) and high MAR (R3, MAR ≥ 1200 mm y^−1^).

MAR range	Vegetation cover	Best model	R^2^
R1-Low MAR	Tree	logit(T) = −3.85 + 0.55·MAR	0.22
	Grass	logit(G) = 0.22–0.42·SI + 0.38·λ_w_	0.56
R2-Intermediate MAR	Tree	logit(T) = −1.48 + 0.49·λ_w_ + 0.14·λ_w_^2^	0.38
	Grass	logit(G) = 0.73–0.17·log_10_(AFI)−0.17·λ_w_	0.37
R3-High MAR	Tree	logit(T) = −1.62–0.41·α_w_	0.32

Predictors are: MAR, rainfall seasonality index (SI), logarithmic average fire interval (log_10_(AFI)), wet-season daily rainfall intensity (α_w_) and wet-season daily rainfall frequency λ_w_. Predictor variables were standardized such that in the GLMs their coefficient magnitude is a measure of their importance in the model. Best models were those with the smaller Akaike information criterion (AIC), see Supplementary Tables S2–S6. No significant model was found for grass cover in R3. The explained deviance (R^2^) is reported for each case. See Material and Methods in the main text for a detailed description of the statistical models and selection procedures.

Specifically, T had a positive trend with MAR (R^2^ = 0.22), which is the most important factor among those we considered in explaining tree cover variations in this range. The inclusion of other rainfall variables in models with MAR only increased the explained deviance by a small amount (ca. 0.04–0.05) (Supplementary Table S2). Tree cover decreased with λ_w_ and SI, and increased with α_w_, however, all of the selected models had an AIC close to the intercept-only model, indicating that they were weak.

**Figure 2:**
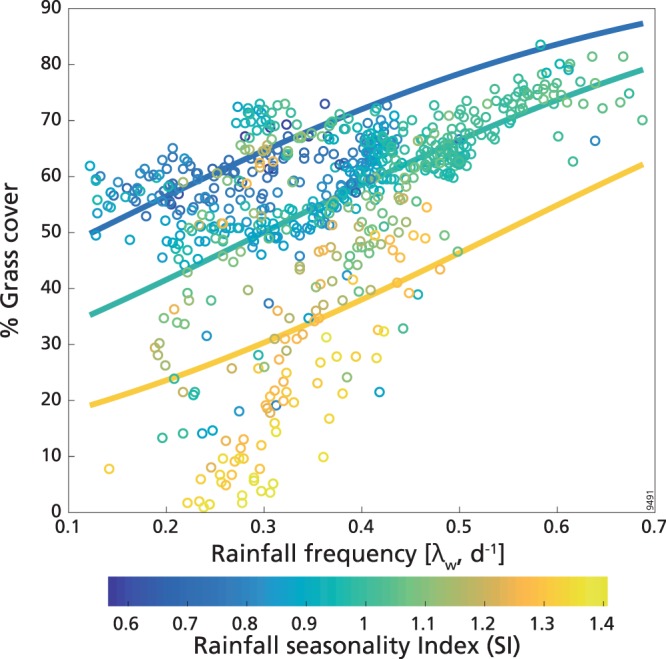
Percentage cover of grass in low mean annual rainfall range (MAR≤630 mm y^−1^) as a function of wet-season rainfall frequency (λ_w_). Colours indicate the seasonality index (SI): for increasing values of SI rainfall occurs in less months during the year. Continuous lines are the best model fit for grass (see Table 1) computed with the median value of SI (equal to 0.97, central line), the 95^th^ percentile (equal to 1.3, lower line) and the 5^th^ percentile (equal to 0.72, higher line).

According to the best model, G, which ranges from ca. 30% to ca. 80% (Fig. 1a), decreased with SI, i.e. it was larger where precipitation was less seasonal, and increased with λ_w_, that is, it was larger where rainfall events were more frequent (Fig. 2). The magnitude of the predictor’s standardized coefficients was similar, and this model could explain 56% of the deviance (see Table 1 and Supplementary Table S3). Although λ_w_ was present in the best model, its effective role on G variations should be analysed in the light of its collinearity with other predictors, and in particular with MAR (r = 0.73, Supplementary Table S1). This was evident from the analyses of the following models and of the residuals. The best models with SI and MAR alone (cubic logit fit, ΔAIC = 4.10, and linear logit fit, ΔAIC = 10.86, respectively, Supplementary Table S3) could explain a much larger fraction of the deviance (53% and 40%, respectively) than the best model with λ_w_ alone (parabolic logit fit, ΔAIC = 26.28, R^2^ = 0.23; in the following we will use ‘parabolic fit’ to indicate ‘parabolic logit fit’). The other explanatory variables (log_10_(AFI) and α_w_) had smaller importance (Supplementary Table S3).

From the residual analysis (see Supplementary Note S1) we found that the linear relationship between the residuals of λ_w_ and G was associated with a small correlation coefficient (R^2^ = 0.07). This indicated that the precipitation frequency variable did not have an important role in determining G without the influence of the other predictors. In fact, λ_w_, as expected, was highly determined by MAR, SI and log_10_(AFI) (in order of importance), which together explained 69% of the deviance for λ_w_ (Supplementary Table S9). Therefore, the increase of G with λ_w_ could be interpreted as due to the simultaneous changes of the other factors: in particular, λ_w_, and consequently G, was higher in areas with higher MAR (which had the largest coefficient in the model).

### 630 mm y^−1^ < MAR < 1200 mm y^−1^

**Figure 3:**
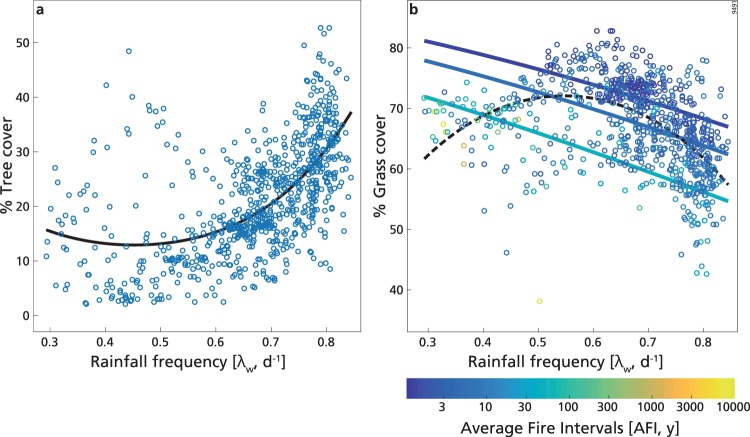
Percentage cover of tree (**a**) and grass (**b**) in the intermediate mean annual rainfall range (630 mm y^−1^ < MAR < 1200 mm y^−1^) as a function of wet-season rainfall frequency (λ_w_). (**a**) Continuous line is the best model fit (see Table 1). (**b**) Continuous lines are the best model fit for grass cover (see Table 1) computed with the median value of AFI (equal to 5 y, central line), the 95^th^ percentile (equal to 47 y, lower line) and the 5^th^ percentile (equal to 1.3 y, higher line). Colours indicate the average fire intervals (AFI). Dashed line indicates the fit of the best model between grass cover and λ_w_ (Supplementary Table S5).

For intermediate precipitation, the best model for T included only λ_w_, through a parabolic fit that explained 38% of the deviance (Fig. 3a, Table 1), while G was mostly explained by log_10_(AFI) and λ_w_ together (R^2^ = 0.37, Table 1, Fig. 3b), showing an opposite dependence on λ_w_ with respect to the low rainfall range. The decreasing trend of grass cover with log_10_(AFI) in this range confirmed the findings of ref.^5^, whose results are comparable in this range as the vast majority of the areas with such MAR in sub-Saharan Africa are covered by TGBs.

According to the best model shown in Fig. 3a, T, which in this range had the larger variation with respect to the other two MAR ranges (Fig. 1a), increased with daily rainfall frequency from 0.45 d^−1^ (obtained from the minimum of the parabolic fit) to ca. 0.85 d^−1^, but varied less for λ_w_ below 0.45 d^−1^ (Fig. 3a). λ_w_ was present in the first 10 models (ΔAIC < 3), in which it always had greater importance than the other variables (Supplementary Table S4). Looking at the contribution of the other factors, T generally increased with MAR, log_10_(AFI), SI and decreased with α_w_ (Supplementary Table S4)_._ However, the dependence of T on these variables alone was very weak (i.e. they had an AIC close to the intercept-only model), and generally explained low deviance (R^2^ ≤ 0.17; Supplementary Table S4), in line with findings from ref.^5^ (though for MAR, log_10_(AFI) and SI only).

According to the best model, G decreased with rainfall frequency, and (similarly to ref.^5^) decreased with the average fire intervals (log_10_(AFI)), i.e. it was favoured by increasing fire frequency (Fig. 3b, Table 1). The model with λ_w_ alone (parabolic fit, third selected model with ΔAIC = 1.72, R^2^ = 0.27, Supplementary Table S5) indicated that G was disadvantaged by increasing precipitation frequency only for λ_w_ greater than 0.55 d^−1^ (obtained from the maximum of the parabolic fit, Fig. 3b), whereas the opposite occurred for lower λ_w_ (Fig. 3b). Interestingly, the maximum of the parabolic fit between G and λ_w_ was relatively close to the minimum of the parabolic fit of T with λ_w_. However, the dependence of G on λ_w_ alone was quite weak (AIC close to the intercept-only model), and this was the case also for the other selected models. SI and MAR were present in models with AIC smaller than the intercept-only model but gave negligible contributions to the explained deviance (Supplementary Table S5).

Through the residual analysis (Supplementary Note S1), we verified that the tendencies of both T and G to increase or decrease in the two parts of their parabolic fits with λ_w_ were independent from MAR, SI and log_10_(AFI), as expected also from the smaller correlation coefficients between predictors in R2 (Supplementary Table S1). Thus, the positive dependence of T on λ_w_ (for λ_w_ > 0.45 d^−1^) and the negative one of G (for λ_w_ > 0.55 d^−1^) were valid for every value of the other explanatory variables and the correlations with the residuals of λ_w_ of the residuals of T was R^2^ = 0.36 and of the residuals of G was R^2^ = 0.31 (Supplementary Fig. S2).

### MAR≥1200 mm y^−1^

**Figure 4:**
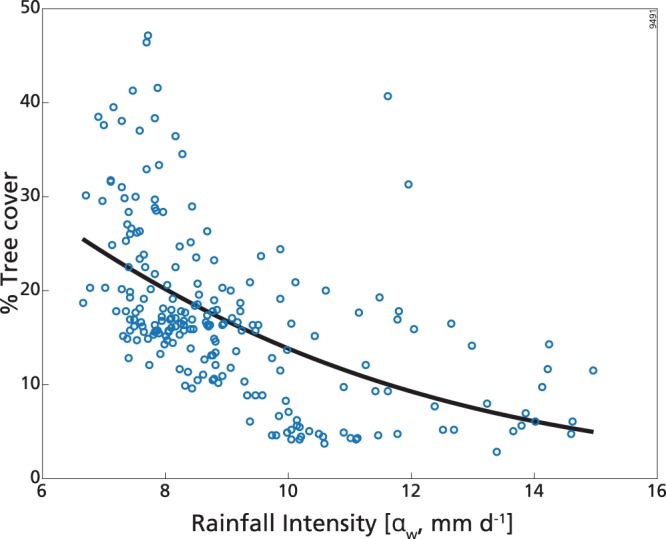
Percentage cover of tree in the high mean annual rainfall range (MAR≥1200 mm y^−1^) as a function of wet-season rainfall intensity (α_w_). Continuous line is the best model fit (see Table 1).

At high precipitation, we found that T mainly depended on α_w_ (Fig. 4), while G was not explained by any explanatory variable (Table 1).

α_w_ was the factor determining most of the variation of T, with decreases in the variable associated with increasing rainfall intensity (Fig. 4, Table 1). This dependence could explain 32% of the deviance for T. There was also a tendency of T to decrease with log_10_(AFI), but this dependence was very weak (Supplementary Table S6). Models with MAR, SI and λ_w_ had AIC greater than the intercept-only model, thus they didn’t play any role.

After removing the influence of MAR, SI and log_10_(AFI) from both T and α_w_, we found that the decreasing of T with α_w_ was preserved, although the importance of this dependence decreased (R^2^ = 0.20, see Supplementary Fig. S2). In fact, the variation of α_w_ was highly determined by SI, MAR and log_10_(AFI) (in order of importance, R^2^ = 0.66, Supplementary Table S9), as expected also from the correlation coefficients between predictors (Supplementary Table S1). Therefore, T decreased with α_w_ partially as a consequence of the simultaneously increase of the latter with MAR, SI and log_10_(AFI). In other words, T was smaller in areas with higher α_w_ partly because they corresponded to areas of higher MAR, higher rainfall seasonality and rare fires.

## Discussion

Our results appear to indicate that intra-seasonal rainfall variability has a role to play in determining the relative predominance of grasses and trees in TGBs in sub-Saharan Africa, with that role and importance dependent on mean annual rainfall (MAR) levels. At the low and intermediate rainfall ranges, the frequency of rainfall during the rainy season was included in the best models explaining grass cover, yet with opposite effects: grasses show a positive (although noisy) trend with rainfall frequency at the dry end of the gradient, while they show a negative trend with it in mesic TGBs, which is especially evident for the highest rainfall frequencies. At this intermediate rainfall level, tree cover also increased with average rainfall frequency in the wet season for the most part, and, somewhat analogously, in the humid TGBs tree cover decreased with rainfall intensity. Importantly, average rainfall frequency in the wet season was shown to explain a reasonable share of the deviance at the intermediate rainfall level for grasses and trees for the first time. The relative importance of intra-seasonal rainfall variability is somewhat lost when investigating the vegetation responses for all TGBs across the sub-continent, with MAR the dominant factor for trees, and seasonality and average fire interval most important for grasses. This highlights the importance of disaggregating the results by MAR range, as the main mechanisms governing TGB dynamics vary with absolute rainfall values^5,7,9^. Differing responses of grass and tree cover to intra-seasonal rainfall variability at different rainfall levels seem to explain the contrasting findings in relation to intra-seasonal variability highlighted by previous research^38,42^.

For grasses, the positive relationship between rainfall frequency and grass cover at the lower MAR level is similar to earlier modelling results for dry savannas^25^. Given that increases in rainfall frequency can enhance water availability in surface soils^19,64^, our finding is consistent with observations regarding the ability of grasses to extract shallow soil water in comparison to trees^65–67^. At lower rainfall levels, the difference between grass root depth and tree root depth is large^18,68^, suggesting a more shallow soil moisture profile would favour grasses, as they also have a more aggressive uptake strategy with respect to trees in dry savannas^16,69^. This result is in line with earlier experimental work^38^, based on a study site with MAR of 544 mm y^−1^, where it was found that increases in intensity and, by virtue of the experimental design, reduction in rainfall frequency, push soil moisture deeper into the soil, benefitting trees with deeper roots at the expense of shallow rooted grasses. In line with this, we also observed, as in other studies^9,36^, that in dry TGBs trees, which are deeper rooted than grasses at this MAR range, increased in cover with more intense, less frequent rainfall. Trees have also access to deeper water during the dryer periods of the year^66^, and increased rainfall intensity with reduced rainfall frequency would assist in enhancing the competitive advantage of trees by increasing deep water recharge. However, it must be noted that intra-seasonal rainfall variables explained a very small part of the deviance for tree cover, which mainly depended on MAR, possibly indicating that tree growth is generally water-limited in these areas. Furthermore, our residual analysis showed that part of the deviance for grass cover explained by rainfall frequency at the lower MAR range is in fact due to its collinearity with the other independent variables, especially with rainfall seasonality and annual amounts. Rainfall seasonality plays a large role at the low MAR range, explaining a large share of the variation for grass cover on its own (R^2^ = 0.53). This implies that of the two variables included in the best model for grasses, seasonality is by far more important than rainfall frequency. It is also important to note that rainfall frequency is also closely correlated with MAR (Pearson’s r = 0.73), and a model including MAR alone explains 40% of the deviance for the grass cover; this highlights the importance, in this low range, of the absolute levels of the water resource for both grasses and trees, as already observed for dry sub-Saharan biomes in general^5^. This may also help to explain why it is rainfall frequency, as opposed to rainfall intensity, that is included in the best model for grass cover, as rainfall intensity shows a much lower correlation with MAR in this range (Pearson’s r = 0.14).

In contrast to the findings for grass cover in the low MAR range, in the intermediate rainfall range, increases in rainfall frequency in the rainy season were associated with a reduction in grass cover for average rainfall frequencies above 0.55 d^−1^. Contrasting responses along a rainfall gradients have been observed previously for grasses in North American prairies^70^, and recently for trees in African TGBs^9^ and across the global tropics^36^. This tendency of grassland to prefer less frequent rainfall at this range is similar to that observed in few experimental field sites in Africa^71^ as well as in other parts of the globe (e.g. ref.^72^). In this intermediate rainfall range, we also observe a positive relationship between tree cover and rainfall frequency (above 0.45 d^−1^), confirming previous results^9,36,42^. While TGB grass cover has been largely unexplained so far in this intermediate range (Supplementary Table S5), more than a quarter of the deviance for grass cover is explained by rainfall frequency alone (R^2^ = 0.27). As previously mentioned, in these mesic savannas there is greater overlap between tree and grass root depth compared to xeric savannas^16,18^, hypothetically implying competition between grasses and mature trees for shallow soil water. The broadly positive relationship between tree cover and rainfall frequency may indicate that trees can exploit surface soil water better than grasses at this MAR range, leading to tree dominance and out-competition of shade-intolerant grasses. This is an interesting finding in our opinion, suggesting that, differently from dry savannas, tree access to deep water is not as relevant in mesic savannas, as also suggested by field observations of ref.^18^, although it is important to temper this finding with reference to the relatively lower explanatory power of rainfall frequency on vegetation cover at this range.

Whereas rainfall frequency in the wet season is included as a predictor in the best models in the low and intermediate ranges, in the higher MAR range increasing rainfall intensity appears to be negatively correlated with tree cover, aligned with earlier findings in relation to rainfall intensity and tree cover^9,42^. Indeed, rainfall intensity is the only variable included in the best model for TGB tree cover in this range. As there is a negative correlation between rainfall frequency and intensity in this MAR range (Pearson’s r = −0.41), this result is also in line with what has been observed at intermediate MAR (for the most part), and with recent findings by ref.^36^, i.e. increases in daily rainfall frequency are associated with increases in tree cover. However, although rainfall intensity remains the best predictor for tree cover, its decrease with rainfall intensity is also partially due to the simultaneous increase of rainfall intensity with both MAR, SI and log10(AFI), as shown by the residual analysis. Hence the signal identified linking rainfall intensity with tree cover is partly noisy.

Overall, although our results support a role for intra-seasonal rainfall variability for trees and grasses in different MAR ranges, our understanding of the mechanisms that lead to vegetation responses is still necessarily limited and speculative. Soil hydrology is complex and an approach taking into account its dynamics explicitly would be needed to proceed further. To understand the benefits to plants of the temporal distribution of rainfall, soil water availability is key, and this depends on a series of factors that also feedback to the vegetation itself, forming a complex system. Although more rare events can be beneficial for vegetation at low precipitation and less beneficial at high precipitation^22^, the picture here is complicated by water competition between trees and grasses, which should be properly disentangled by including the changes in dynamics along a rainfall gradient, for example including the MAR-dependent relative rooting depth of tree and grasses^18^. Tree access to deep water during the dry season may generate long-term memory effects^73^, and contribute to different phenology of trees and grasses^74^, which can lead to a temporal niche-partitioning^48^, a known mechanism of species coexistence (the so-called storage-effect)^44^. Soil texture influences water availability, and it thus mediates the response of tree cover to intra-seasonal rainfall variability, with a role that can be almost as important as total yearly rainfall^9,19^. Finally, we would like to acknowledge that the scale of the hydrological dynamics of tree-grass water competition is much finer than the scale of the data used in the present study (0.5°). Given this resolution, the intra-seasonal rainfall variables here considered displayed very small variations in their mean values. However, these variations are seemingly relevant for vegetation, with local relationship possibly scaling up and emerging at the coarser scale of this analysis.

TGBs are likely to show contrasting responses to anthropogenic climate change, which is expected to increase rainfall intensity across Africa^35^, depending on the absolute values of mean annual rainfall. If this increase will be accompanied by a decrease of rainfall frequency, our results suggest that grasses will decrease in dry TGBs possibly favouring further woody encroachment, while woody cover will be limited in mesic and humid savannas. Our findings suggest that intra-seasonal rainfall variability is especially important for grasses and trees in mesic TGBs. For these areas in particular, our research serves to enhance the so-far-limited understanding of the factors determining the relative balance of these two vegetation life-forms.

## Supplementary information

Supplementary Information

## Data Availability

The observational datasets used in this study are all freely available. The TRMM 3B42 dataset is available at https://mirador.gsfc.nasa.gov/. The ESA CCI-LC, v 1.6.1 dataset is available at http://maps.elie.ucl.ac.be/CCI/viewer/download.php. The MODIS datasets (MOD44B and MOD45A1) are available at https://earthdata.nasa.gov. Post-processed data are available upon request to the authors.
