# Supplementary material for: Grass and tree cover responses to intra-seasonal rainfall variability vary along a rainfall gradient in African tropical grassy biomes
Source: Sci Rep. 2019 Feb 20;9:2334. doi: 10.1038/s41598-019-38933-9 (PMC6382848; doi:10.1038/s41598-019-38933-9)
Supplement: Supplementary file 1 — Supplementary Information [file 41598_2019_38933_MOESM1_ESM.pdf]

Supplementary Information

Article title: Grass and tree cover responses to intra-seasonal rainfall variability vary along a rainfall gradient in African tropical grassy biomes

Authors: Donatella D'Onofrio, Luke Sweeney, Jost von Hardenberg and Mara Baudena

List of contents:

Figure S1: Map of the spatial distribution of the three mean annual rainfall ranges
Table S1: Pearson's r coefficients between explanatory variables in the three mean annual rainfall ranges
Table S2: Generalized linear models for tree cover in the low mean annual rainfall range
Table S3: Generalized linear models for grass cover in the low mean annual rainfall range
Table S4: Generalized linear models for tree cover in the intermediate mean annual rainfall range
Table S5: Generalized linear models for grass cover in the intermediate mean annual rainfall range
Table S6: Generalized linear models for tree cover in the high mean annual rainfall range
Table S7: Generalized linear models for tree cover without filtering by mean annual rainfall ranges
Table S8: Generalized linear models for grass cover without filtering by mean annual rainfall ranges
Note S1: Supplementary information for the residual analysis. Method and Results
Table S9: Generalized linear models for the residual analysis
Figure S2: Results of the residual analysis

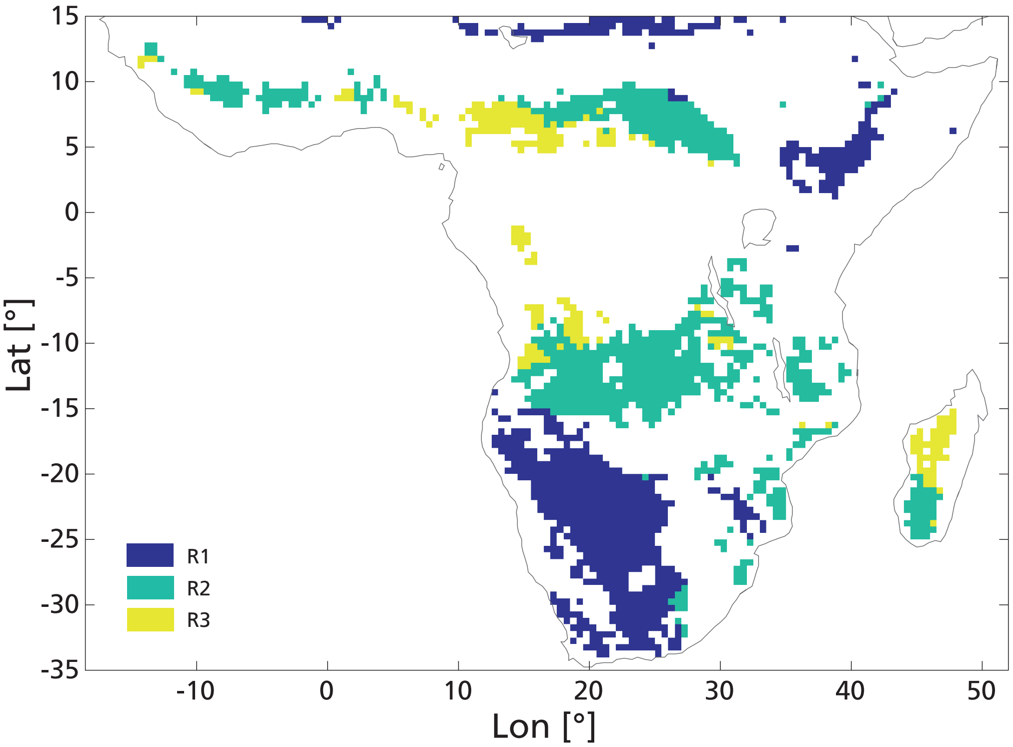

Figure S1: Geographical distribution of 0.5° grid cells in areas of tropical grassy biomes in sub-Saharan Africa (determined as explained in the Material and Methods section in the main text) within the three mean annual rainfall ranges R1 (0-630 mm y^{-1}), R2 (630-1200 mm y^{-1}) and R3 (1200-2500 mm y^{-1}).

Table S1: Pearson's r coefficients between explanatory variables in the three mean annual rainfall (MAR) ranges. Pearson's r between MAR, rainfall seasonality index (SI), logarithmic average fire intervals (log_{10}(AFI)), wet-season rainfall intensity (α_w) and frequency (λ_w) for R1 (MAR≤630 mm y^{-1}), R2 (630 mm y^{-1} <MAR < 1200 mm y^{-1}) and R3 (MAR ≥ 1200 mm y^{-1})

**MAR range**		**MAR**	**SI**	**Log_{10}(AFI)**	α_w	λ_w
R1- Low MAR	**MAR**	1	-0.26	-0.64	0.14	0.73
	**SI**	-0.26	1	-0.1	0.27	0.16
	**log_{10}(AFI)**	-0.64	-0.10	1	-0.01	-0.67
	α_w	0.14	0.27	-0.01	1	-0.30
	λ_w	0.73	0.16	-0.67	-0.30	1
R2- Intermediate MAR	**MAR**	1	-0.14	-0.21	0.33	0.25
	**SI**	-0.14	1	0.15	0.19	0.33
	**log_{10}(AFI)**	-0.21	0.15	1	0.30	-0.33
	α_w	0.33	0.19	0.30	1	-0.60
	λ_w	0.25	0.33	-0.33	-0.60	1
R3-High MAR	**MAR**	1	-0.24	0.11	0.41	0.31
	**SI**	-0.24	1	0.23	0.57	-0.06
	**log_{10}(AFI)**	0.11	0.23	1	0.34	-0.17
	α_w	0.41	0.57	0.34	1	-0.41
	λ_w	0.31	-0.06	-0.17	-0.41	1

Table S2: GLMs for tree cover in the low mean annual rainfall range (MAR≤630 mm y^{-1}). Explanatory variables are: MAR, rainfall seasonality index (SI), logarithmic average fire interval (log_{10}(AFI)), wet-season daily rainfall intensity (α_w) and wet-season rainfall frequency (λ_w). Among all the possible combinations of predictors, only models with Akaike information criterion (AIC) smaller than the intercept-only model are shown. For each case we report the coefficients of the predictors (x1-x4), the explained deviance (R^2) and the AIC differences (ΔAIC). Note that predictor variables were standardized such that in the GLMs their coefficient magnitude is a measure of their importance in the model. See Material and Methods in the main text for a detailed description of the statistical model analysis.

**x1**	**x2**	**x3**	**x4**	**Coef Itercept**	**Coef x1**	**Coef x2**	**Coef x3**	**Coef x4**	**ΔAIC**	**R^2**
MAR	/	/	/	-3.85	0.55	/	/	/	0	0.22
MAR	λ_w	/	/	-3.87	0.77	-0.34	/	/	0.93	0.27
MAR	MAR^2	/	/	-4.07	0.50	0.22	/	/	1.14	0.26
MAR	SI	/	/	-3.88	0.49	-0.26	/	/	1.21	0.26
MAR	α_w	/	/	-3.87	0.52	0.18	/	/	1.37	0.25
SI	log_{10}(AFI)	/	/	-3.86	-0.44	-0.42	/	/	1.83	0.23
MAR	log_{10}(AFI)	/	/	-3.85	0.53	-0.03	/	/	1.99	0.22
SI	log_{10}(AFI)	α_w	/	-3.90	-0.49	-0.42	0.32	/	1.99	0.32
/	/	/	/	-3.72	/	/	/	/	2.12	0

Table S3: Same as Table S2 but GLMs are for grass cover in the low mean annual rainfall range (MAR≤630 mm y^{-1}).

x1	x2	x3	x4	Coef Itercept	Coef x1	Coef x2	Coef x3	Coef x4	ΔAIC	R^2
SI	λ_w		/	0.22	-0.42	0.38	/	/	0	0.56
MAR	SI	λ_w	/	0.22	0.17	-0.36	0.25	/	0.53	0.57
SI	$\log_{10}$(AFI)	λ_w	/	0.22	-0.42	-0.05	0.35	/	1.79	0.56
MAR	SI	/	/	0.22	0.38	-0.27	/	/	1.80	0.53
MAR	SI	α_w	/	0.22	0.40	-0.23	-0.12	/	1.84	0.56
MAR	SI	$\log_{10}$(AFI)	λ_w	0.22	0.17	-0.36	/	0.25	2.53	0.57
MAR	SI	$\log_{10}$(AFI)	/	0.22	0.34	-0.29	-0.05	/	3.57	0.54
MAR	SI	$\log_{10}$(AFI)	α_w	0.22	0.39	-0.24	-0.02	-0.11	3.82	0.56
SI	SI^2	SI^3	/	0.45	0.03	-0.21	-0.12	/	4.10	0.53
MAR	α_w	/	/	0.22	0.47	-0.19	/	/	7.06	0.47
SI	SI^2	/	/	0.45	-0.27	-0.25	/	/	7.32	0.46
MAR	$\log_{10}$(AFI)	α_w	/	0.22	0.53	0.10	-0.20	/	8.13	0.48
SI	$\log_{10}$(AFI)	/	/	0.22	-0.39	-0.28	/	/	10.48	0.42
MAR	/	/	/	0.22	0.44	/	/	/	10.86	0.40
MAR	$\log_{10}$(AFI)	/	/	0.22	0.49	0.07	/	/	12.34	0.40
SI	$\log_{10}$(AFI)	α_w	/	0.22	-0.39	-0.28	-0.03	/	12.38	0.43
MAR	MAR^2	/	/	0.20	0.45	0.02	/	/	12.76	0.40
MAR	λ_w	/	/	0.22	0.46	-0.02	/	/	12.82	0.40
MAR	$\log_{10}$(AFI)	λ_w	/	0.22	0.48	0.08	0.01	/	14.33	0.40
MAR	MAR^2	MAR^3	/	0.21	0.50	/	-0.03	/	14.58	0.40
SI	/	/	/	0.22	-0.36	/	/	/	21.22	0.27
SI	α_w	/	/	0.21	-0.35	-0.03	/	/	23.08	0.27
λ_w	λ_w^2	/	/	0.13	0.30	0.09	/	/	26.28	0.23
λ_w	/	/	/	0.22	0.31	/	/	/	26.35	0.20
λ_w	λ_w^2	λ_w^3	/	0.11	0.43	0.12	-0.05	/	27.08	0.24
$\log_{10}$(AFI)	λ_w	/	/	0.22	-0.05	0.28	/	/	28.13	0.20
$\log_{10}$(AFI)	α_w	/	/	0.21	-0.24	-0.13	/	/	32.53	0.15
$\log_{10}$(AFI)	$\log_{10}(AFI)^2$	/	/	0.10	-0.24	0.12	/	/	33.16	0.14
$\log_{10}$(AFI)	/	/	/	0.21	-0.23	/	/	/	33.23	0.12
$\log_{10}$(AFI)	$\log_{10}(AFI)^2$	$\log_{10}(AFI)^3$	/	0.10	-0.20	0.11	-0.02	/	35.10	0.14
α_w	/	/	/	0.21	-0.12	/	/	/	39.90	0.03
/	/	/	/	0.21	/	/	/	/	40.44	0

Table S4: Same as Table S2 but GLMs are for tree cover in the intermediate mean annual rainfall range (630 mm y^{-1}<MAR<1200 mm y^{-1}).

**x1**	**x2**	**x3**	**x4**	**Coef Itercept**	**Coef x1**	**Coef x2**	**Coef x3**	**Coef x4**	**ΔAIC**	**R^2**
λ_w	λ_w^2	/	/	-1.48	0.49	0.14	/	/	0	0.38
λ_w	/	/	/	-1.34	0.38	/	/	/	0.93	0.32
$\log_{10}$(AFI)	λ_w	/	/	-1.34	0.13	0.41	/	/	1.10	0.35
MAR	$\log_{10}$(AFI)	λ_w	/	-1.35	0.13	0.15	0.39	/	1.34	0.39
MAR	λ_w	/	/	-1.34	0.11	0.36	/	/	1.62	0.34
λ_w	λ_w^2	λ_w^3	/	-1.46	0.52	0.10	-0.02	/	1.92	0.38
MAR	SI	λ_w	/	-1.35	0.14	0.12	0.30	/	2.08	0.37
SI	λ_w	/	/	-1.34	0.08	0.35	/	/	2.17	0.33
MAR	SI	$\log_{10}$(AFI)	λ_w	-1.35	0.15	0.09	0.13	0.34	2.64	0.40
SI	$\log_{10}$(AFI)	λ_w	/	-1.34	0.05	0.12	0.39	/	2.87	0.36
MAR	SI	α_w	/	-1.34	0.30	0.29	-0.21	/	6.24	0.29
MAR	SI	$\log_{10}$(AFI)	α_w	-1.34	0.33	0.28	0.10	-0.25	7.30	0.31
MAR	SI	/	/	-1.32	0.22	0.23	/	/	8.22	0.21
MAR	SI	$\log_{10}$(AFI)	/	-1.32	0.22	0.23	0.02	/	10.18	0.21
MAR	MAR^2	/	/	-1.13	0.14	-0.19	/	/	10.21	0.17
SI	/	/	/	-1.31	0.19	/	/	/	11.96	0.10
MAR	MAR^2	MAR^3	/	-1.13	0.09	-0.18	0.03	/	12.12	0.17
MAR	/	/	/	-1.31	0.18	/	/	/	12.56	0.08
$\log_{10}$(AFI)	$\log_{10}(AFI)^2$	/	/	-1.19	0.17	-0.14	/	/	12.92	0.12
MAR	α_w	/	/	-1.31	0.22	-0.12	/	/	13.16	0.11
SI	α_w	/	/	-1.31	0.21	-0.08	/	/	13.25	0.11
MAR	$\log_{10}$(AFI)	α_w	/	-1.31	0.26	0.11	-0.16	/	13.85	0.14
SI	$\log_{10}$(AFI)	/	/	-1.31	0.20	-0.02	/	/	13.92	0.10
SI	SI^2	/	/	-1.30	0.19	-0.01	/	/	13.94	0.10
MAR	$\log_{10}$(AFI)	/	/	-1.31	0.19	0.05	/	/	14.23	0.09
$\log_{10}$(AFI)	$\log_{10}(AFI)^2$	$\log_{10}(AFI)^3$	/	-1.15	0.15	-0.20	0.02	/	14.40	0.13
/	/	/	/	-1.30	/	/	/	/	14.67	0

Table S5: Same as Table S2 but GLMs are for grass cover in the intermediate mean annual rainfall range (630 mm y^{-1}<MAR<1200 mm y^{-1}).

x1	x2	x3	x4	Coef Itercept	Coef x1	Coef x2	Coef x3	Coef x4	ΔAIC	R^2
$\log_{10}$(AFI)	λ_w	/	/	0.73	-0.17	-0.17	/	/	0	0.37
SI	$\log_{10}$(AFI)	λ_w	/	0.73	-0.05	-0.16	-0.15	/	1.64	0.39
λ_w	λ_w^2	/	/	0.84	-0.22	-0.11	/	/	1.72	0.27
MAR	$\log_{10}$(AFI)	λ_w	/	0.73	-0.03	-0.18	-0.16	/	1.84	0.38
SI	/	/	/	0.72	-0.12	/	/	/	2.15	0.14
$\log_{10}$(AFI)	/	/	/	0.72	-0.12	/	/	/	2.31	0.13
SI	$\log_{10}$(AFI)	/	/	0.72	-0.11	-0.10	/	/	2.33	0.24
λ_w	/	/	/	0.72	-0.11	/	/	/	2.71	0.11
/	/	/	/	0.72	/	/	/	/	2.77	0

Table S6: Same as Table S2 but GLMs are for tree cover in the high mean annual rainfall range (MAR≥1200 mm y^{-1}).

x1	x2	x3	x4	Coef Itercept	Coef x1	Coef x2	Coef x3	Coef x4	ΔAIC	R^2
α_w	/	/	/	-1.62	-0.41	/	/	/	0	0.32
$\log_{10}$(AFI)	α_w	/	/	-1.62	-0.16	-0.35	/	/	1.30	0.38
α_w	α_w^2	/	/	-1.74	-0.54	0.13	/	/	1.44	0.37
$\log_{10}$(AFI)	/	/	/	-1.59	-0.27	/	/	/	1.73	0.18
/	/	/	/	-1.57	/	/	/	/	1.87	0

Table S7: Same as Table S2 but GLMs are for tree cover without filtering by mean annual rainfall ranges.

x1	x2	x3	x4	Coef Itercept	Coef x1	Coef x2	Coef x3	Coef x4	ΔAIC	R^2
MAR	MAR^2	MAR^3	/	-1.58	1.16	-0.97	0.18	/	0	0.67
MAR	MAR^2	/	/	-1.76	1.24	-0.57	/	/	6.39	0.64
$log_{10}(AFI)$	λ_w	/	/	-2.27	-0.25	0.88	/	/	10.70	0.62
λ_w	/	/	/	-2.23	1.01	/	/	/	11.59	0.60
MAR	$log_{10}(AFI)$	λ_w	/	-2.27	0.06	-0.24	0.83	/	12.36	0.62
SI	$log_{10}(AFI)$	λ_w	/	-2.27	-0.03	-0.23	0.89	/	12.54	0.62
SI	λ_w	/	/	-2.24	-0.07	1.01	/	/	13.00	0.61
MAR	λ_w	/	/	-2.23	0.08	0.95	/	/	13.11	0.61
λ_w	λ_w^2	λ_w^3	/	-2.30	1.28	0.02	-0.16	/	13.48	0.61
λ_w	λ_w^2	/	/	-2.24	1.01	0.01	/	/	13.58	0.60
MAR	SI	$log_{10}(AFI)$	λ_w	-2.27	0.06	-0.01	-0.24	0.84	14.35	0.62
MAR	SI	λ_w	/	-2.24	0.04	-0.05	0.98	/	14.88	0.61
MAR	SI	$log_{10}(AFI)$	α_w	-2.20	0.68	0.26	-0.55	-0.22	38.89	0.51
MAR	SI	$log_{10}(AFI)$	/	-2.20	0.48	0.18	-0.64	/	40.80	0.49
MAR	$log_{10}(AFI)$	/	/	-2.18	0.41	-0.63	/	/	42.71	0.48
MAR	$log_{10}(AFI)$	α_w	/	-2.17	0.48	-0.59	-0.09	/	43.77	0.48
$log_{10}(AFI)$	$log_{10}(AFI)^2$	$log_{10}(AFI)^3$	/	-2.01	-1.54	-0.56	0.37	/	49.98	0.45
MAR	SI	α_w	/	-2.10	1	0.31	-0.38	/	54.73	0.43
$log_{10}(AFI)$	$log_{10}(AFI)^2$	/	/	-1.96	-0.90	-0.31	/	/	56.43	0.41
$log_{10}(AFI)$	α_w	/	/	-2.15	-0.88	0.14	/	/	58.49	0.41
$log_{10}(AFI)$	/	/	/	-2.13	-0.89	/	/	/	60.40	0.39
SI	$log_{10}(AFI)$	α_w	/	-2.15	0.01	-0.88	0.14	/	60.48	0.41
SI	$log_{10}(AFI)$	/	/	-2.14	0.02	-0.89	/	/	62.34	0.39
MAR	α_w	/	/	-2.06	0.79	-0.23	/	/	63.32	0.38
MAR	SI	/	/	-2.05	0.70	0.17	/	/	65.73	0.37
MAR	/	/	/	-2.04	0.64	/	/	/	67.56	0.36
α_w	α_w^2	α_w^3	/	-1.62	0.43	-0.5	0.10	/	120.25	0.14
α_w	α_w^2	/	/	-1.80	0.46	-0.14	/	/	128.85	0.09
SI	SI^2	SI^3	/	-1.73	0.18	-0.23	-0.14	/	130.86	0.09
SI	α_w	/	/	-1.92	-0.13	0.23	/	/	136.27	0.06
α_w	/	/	/	-1.91	0.22	/	/	/	137.40	0.05
SI	SI^2	/	/	-1.76	-0.13	-0.16	/	/	139.79	0.05
SI	/	/	/	-1.9	-0.11	/	/	/	145.77	0.01
/	/	/	/	-1.89	/	/	/	/	146.22	0

Table S8: Same as Table S2 but GLMs are for grass cover without filtering by mean annual rainfall ranges.

x1	x2	x3	x4	Coef Itercept	Coef x1	Coef x2	Coef x3	Coef x4	ΔAIC	R^2
SI	$\log_{10}$(AFI)	/	/	0.54	-0.25	-0.27	/	/	0	0.46
SI	$\log_{10}$(AFI)	λ_w	/	0.54	-0.25	-0.20	0.09	/	0.87	0.47
MAR	SI	$\log_{10}$(AFI)	/	0.54	0.06	-0.23	-0.23	/	1.28	0.46
SI	$\log_{10}$(AFI)	α_w	/	0.54	-0.25	-0.26	0.04	/	1.33	0.46
SI	SI^2	SI^3	/	0.65	-0.04	-0.12	-0.07	/	1.43	0.46
MAR	SI	$\log_{10}$(AFI)	λ_w	0.54	0.02	-0.25	-0.20	0.07	2.82	0.47
MAR	SI	$\log_{10}$(AFI)	α_w	0.54	0.04	-0.24	-0.24	0.02	3.16	0.46
SI	λ_w	/	/	0.54	-0.27	0.24	/	/	5.20	0.42
MAR	SI	λ_w	/	0.54	0.05	-0.25	0.20	/	6.92	0.42
MAR	MAR^2	MAR^3	/	0.78	0.17	-0.28	0.09	/	7.26	0.42
SI	SI^2	/	/	0.68	-0.25	-0.15	/	/	8.24	0.39
MAR	SI	/	/	0.54	0.23	-0.21	/	/	9.96	0.38
MAR	SI	α_w	/	0.54	0.27	-0.20	-0.05	/	11.42	0.38
MAR	MAR^2	/	/	0.68	0.35	-0.14	/	/	13.48	0.35
λ_w	λ_w^2	λ_w^3	/	0.80	0.43	-0.30	-0.14	/	16.78	0.34
MAR	$\log_{10}$(AFI)	/	/	0.53	0.17	-0.19	/	/	17.36	0.32
MAR	$\log_{10}$(AFI)	α_w	/	0.53	0.23	-0.17	-0.08	/	17.84	0.34
MAR	$\log_{10}$(AFI)	λ_w	/	0.53	0.20	-0.22	-0.07	/	18.87	0.33
λ_w	λ_w^2	/	/	0.73	0.20	-0.20	/	/	20.46	0.30
$\log_{10}$(AFI)	/	/	/	0.53	-0.30	/	/	/	21.03	0.28
MAR	α_w	/	/	0.53	0.37	-0.12	/	/	21.31	0.29
$\log_{10}$(AFI)	$\log_{10}(AFI)^2$	/	/	0.47	-0.36	0.06	/	/	21.78	0.29
$\log_{10}$(AFI)	λ_w	/	/	0.53	-0.25	0.07	/	/	22.27	0.29
$\log_{10}$(AFI)	α_w	/	/	0.53	-0.30	0.02	/	/	22.89	0.28
MAR	/	/	/	0.53	0.30	/	/	/	22.97	0.27
SI	α_w	/	/	0.53	-0.29	0.11	/	/	23.53	0.28
$\log_{10}$(AFI)	$\log_{10}(AFI)^2$	$\log_{10}(AFI)^3$	/	0.49	-0.40	0.02	0.03	/	23.55	0.29
MAR	λ_w	/	/	0.53	0.24	0.07	/	/	24.28	0.27
SI	/	/	/	0.53	-0.29	/	/	/	25.78	0.24
λ_w	/	/	/	0.53	0.26	/	/	/	30.14	0.21
α_w	/	/	/	0.52	0.09	/	/	/	54.58	0.02
/	/	/	/	0.52	/	/	/	/	55.71	0

Note S1: Supplementary information for the residual analysis. Method and Results

METHOD

In order to understand the effective dependence of the vegetation cover variables on the intra-seasonal rainfall variables (i.e. whether this dependence was or not influenced by other explanatory variables due to collinearity) we performed a residual analysis for the cases where the best models for vegetation cover included the wet-season rainfall intensity (α_w) or frequency (λ_w) (See Table 1 in the main text).

To this end, given V the vegetation cover (tree cover T or grass cover G) and R the intra-seasonal rainfall variables (α_w or λ_w), we computed:

(1) The multi-variable GLM of V with mean annual rainfall (MAR), rainfall seasonality index (SI) and average fire intervals in logarithmic scale ($\log_{10}$(AFI)), included as linear terms (see also Material and Method section in the main text, and Table S2-S6 for the results).
(2) The deviance residuals of the GLM for V (V').
(3) The multi-variable GLM of R with MAR, SI, $\log_{10}$(AFI), included as linear terms (see Table S9 below). λ_w was fitted assuming binomial error distribution with a logit function, because, like vegetation cover, it is limited between 0 and 1, while α_w was fitted assuming normal error distribution.
(4) The deviance residuals of the GLM for R (R').
(5) The linear fit between V' and R', evaluated using the R^2. A high R^2 implied that the dependence of V on R was direct and not only influenced by the other variables.

RESULTS

Table S9 below summarizes the GLMs for α_w or λ_w (see step 3 of the procedure described above) and the GLMs for tree and grass cover for the intermediate rainfall range (see step 1 above), which are not included in Tables S2-S6 for the following reason: in the intermediate range the best GLM for T with λ_w was a parabolic logit fit (Table 1), thus we computed the residual analysis for data below and above the parabolic logit fit minimum (λ_w=0.45 d^{-1}), in order to investigate the effective tendency (i.e. increase or decrease) of tree cover with respect to λ_w. Since also G had a parabolic logit dependence on λ_w , quite specular to trees, we computed, as done for trees, the residual analysis for data below and above the parabolic logit fit maximum (λ_w=0.55 d^{-1}), even though this model was the third selected model (ΔAIC=1.72, see Fig. 3b and Table S5).

Table S9: Generalized linear models for the residual analysis. GLMs for mean daily rainfall intensity in the wet-season (α_w) or mean rainfall frequency in the wet season (λ_w) in the three mean annual rainfall (MAR) ranges: low MAR (R1, MAR≤630 mm y^{-1}), intermediate MAR (R2, 630 mm y^{-1} <MAR < 1200 mm y^{-1}) and high MAR (R3, MAR ≥ 1200 mm y^{-1}). GLMs for tree and grass cover in R2 are also reported. Predictors are: MAR, rainfall seasonality index (SI), logarithmic average fire interval ($\log_{10}$(AFI)). The explained deviance (R^2) is reported for each case. Note that predictor variables were standardized such that in the GLMs their coefficient magnitude is a measure of their importance in the model.

**MAR range**	**Depended variable (Intra-seasonal rainfall variable or vegetation cover variable)**	**GLM**	**R^2**
R1- Low MAR	λ_w (included in the best model for G, see Table 1)	$logit(\lambda_w) = -0.57 + 0.36\cdot MAR + 0.16\cdot SI - 0.10\cdot \log_{10}(AFI)$	0.69
R2-Intermediate MAR	T, λ_w (for λ_w < 0.45 d^{-1}, minimum of the parabolic logit fit for T, see Table 1)	$logit(\lambda_w) = -0.48 + 0.01\cdot MAR - 0.06\cdot SI - 0.09\cdot \log_{10}(AFI)$	0.26
		$logit(T) = -2.03 + 0.40\cdot MAR + 0.36\cdot SI - 0.23\cdot \log_{10}(AFI)$	0.57
	T, λ_w (for λ_w ≥0.45 d^{-1} minimum of the parabolic logit fit for T, see Table 1)	$logit(\lambda_w) = 0.84 + 0.10\cdot MAR + 0.20\cdot SI - 0.06\cdot \log_{10}(AFI)$	0.20
		$logit(T) = -1.28 + 0.17\cdot MAR + 0.17\cdot SI + 0.10\cdot \log_{10}(AFI)$	0.18
	G, λ_w (for λ_w <0.55 d^{-1} maximum of the parabolic logit fit for G, see Fig. 3b and Table S5)	$logit(\lambda_w) = -0.22 - 0.04\cdot MAR - 0.06\cdot SI - 0.12\cdot \log_{10}(AFI)$	0.28
		$logit(G) = 0.78 - 0.10\cdot MAR - 0.15\cdot SI - 0.13\cdot \log_{10}(AFI)$	0.43
	G, λ_w (for λ_w ≥0.55 d^{-1} minimum of the	$logit(\lambda_w) = 0.93 + 0.07\cdot MAR + 0.12\cdot SI - 0.01\cdot \log_{10}(AFI)$	0.14

	parabolic logit fit for G, see Fig. 3b and Table S5)	logit(G) = 0.71 -0.05·MAR -0.07·SI -0.14·$\log_{10}$(AFI)	0.30
R3-High MAR	α_w (included in the best model for T, see Table 1)	α_w=9.0+ 1.02·MAR + 1.23·SI + 0.22·$\log_{10}$(AFI)	0.66

After the computation of the residuals of the GLMs for vegetation cover and for intra-seasonal rainfall variables (see steps 2,4 in the procedure above), we computed the linear fits between these two residual sets (see step 5 in the procedure above). Figure S2 shows the scatterplot of the residuals along with the linear fits and the R^2 for the different MAR ranges.

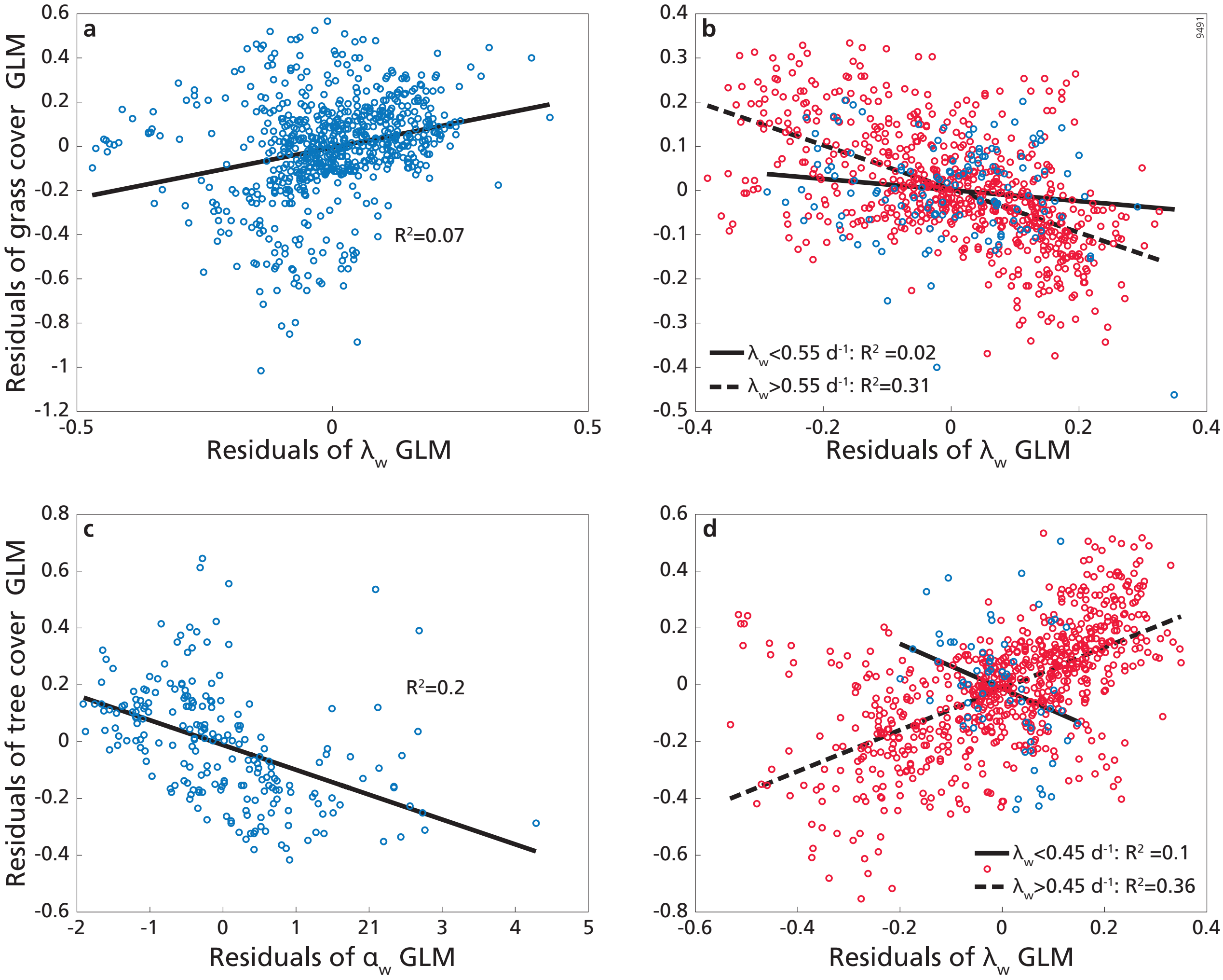

Figure S2: Results of the residual analysis. (a) Scatter plot between the deviance residuals of the grass cover GLM (y-axis) and the deviance residuals of λ_w GLM (x-axis) at low mean annual rainfall (MAR ≤630 mm y^{-1}). Continuous line is the linear fit between the two residual sets; (b) Scatter plot between the deviance residuals of the grass cover GLM (y-axis) and the deviance residuals of λ_w GLM (x-axis) computed for $\lambda_w < 0.55$ d^{-1} (blue circles) and $\lambda_w \geq 0.55$ d^{-1} (red circles) at intermediate mean annual rainfall (630 mm y^{-1} <MAR < 1200 mm y^{-1}). Lines are the linear fits between the two

residual sets for $\lambda_w < 0.55$ d^{-1} (continuous line) and for $\lambda_w \geq 0.55$ d^{-1} (dashed line); (c) Scatter plot between the deviance residuals of the tree cover GLM (y-axis) and the deviance residuals of α_w GLM (x-axis) at high mean annual rainfall (MAR≥630 mm y^{-1}). Continuous line is the linear fit between the two residual sets; (d) Scatter plot between the deviance residuals of the tree cover GLM (y-axis) and the deviance residuals of λ_w GLM (x-axis) computed for $\lambda_w < 0.45$ d^{-1} (blue circles) and $\lambda_w \geq 0.45$ d^{-1} (red circles) at intermediate mean annual rainfall (630 mm y^{-1} <MAR < 1200 mm y^{-1}). Lines are the linear fits between the two residual sets for $\lambda_w < 0.45$ d^{-1} (continuous line) and for $\lambda_w \geq 0.45$ d^{-1} (dashed line). All panels show the R^2 of the linear fits.
